# How Cues of Being Watched Promote Risk Seeking in Fund Investment in Older Adults

**DOI:** 10.3389/fpsyg.2021.765632

**Published:** 2022-01-12

**Authors:** Meijia Li, Huamao Peng

**Affiliations:** ^1^Institute of Developmental Psychology, Beijing Normal University, Beijing, China; ^2^Beijing Key Laboratory of Applied Experimental Psychology, Beijing Normal University, Beijing, China

**Keywords:** risk seeking, eye movement, fund decision making, framing effect, being watched

## Abstract

Social cues, such as being watched, can subtly alter fund investment choices. This study aimed to investigate how cues of being watched influence decision-making, attention allocation, and risk tendencies. Using decision scenarios adopted from the “Asian Disease Problem,” we examined participants’ risk tendency in a financial scenario when they were watched. A total of 63 older and 66 younger adults participated. Eye tracking was used to reveal the decision-maker’s attention allocation (fixations and dwell time per word). The results found that both younger and older adults tend to seek risk in the loss frame than in the gain frame (i.e., framing effect). Watching eyes tended to escalate reckless gambling behaviors among older adults, which led them to maintain their share in the depressed fund market, regardless of whether the options were gain or loss framed. The eye-tracking results revealed that older adults gave less attention to the sure option in the eye condition (i.e., fewer fixations and shorter dwell time). However, their attention was maintained on the gamble options. In comparison, images of “watching eyes” did not influence the risk seeking of younger adults but decreased their framing effect. Being watched can affect financial risk preference in decision-making. The exploration of the contextual sensitivity of being watched provides us with insight into developing decision aids to promote rational financial decision-making, such as human-robot interactions. Future research on age differences still requires further replication.

## Introduction

In our society, older adults face many complicated decisions about financial investment and retirement choices. However, older people make up the vast majority of fraud victims, which makes them targets of financial abuse (Lichtenberg et al., 2016). Behavioral economics research has found that older adults tend to rely more on heuristic processing than younger adults, which hints that older adults are more likely to show framing effects (Kim et al., 2005; Cooper et al., 2017). The presence of others can subtly alter investment choices. Heightened compliance, a sense of being monitored, and increasing reward sensitivity in decision-making when social cues are present could explain the boosted risk-seeking tendency to gain profits (Haley and Fessler, 2005; Weigard et al., 2014; Bittner and Kulesz, 2015).

Eye gaze is considered a rudimentary social signal (Duan et al., 2020). In our daily life, social cues, such as being watched, may alter fund investment choices in the financial market. Social interaction involves complex communication *via* various social signals, such as watching eyes, facial expressions, gestures, and tones. Gaze cues are one of the most informative social stimuli, which reflect social attentional focus (Jessen and Grossmann, 2014; Liu et al., 2021). When we feel that someone is watching us, our behavior will change (Bateson et al., 2006; Karvay et al., 2021). How being watched potentially influences an individual’s fund decision-making is an interesting question in both social psychology and behavioral economics. In the following study, we aim to ascertain whether older adults in fund investment can make “rational” decisions on fund investment, and whether being watched, which functions as a social cue, influences their fund decisions framed as gains or losses.

### Framing Effect on Decision-Making

Risky-choice framing paradigms usually present participants with both sure and gamble options that involve risk. Subtle changes in the wording of decision scenarios have been shown to affect one’s risk preferences (Perez et al., 2018). A reliable manipulation is phrasing the question to emphasize the gain or loss aspect of the equivalent scenario that leads to risk aversion or risk seeking, respectively, which is called the “framing effect.” The most convincing example came from Kahneman and Tversky’s research on the “Asian disease” problem (Tversky and Kahneman, 1981). The participants made different decisions when faced with two effectively identical solutions framed in different ways. The transitivity of preferences provides criteria for the rationality of choices. In contrast to previous experimental tasks, the present study employed a relatively ecological vignette by creating fund decision scenarios.

Fund purchases are one of the most closely related daily decision-making situations related to the framing effect and risky decisions. Fund investment involves gains and losses, which provide proxies to examine risk-averse and risk-seeking tendencies. Following the same logic of the “Asian disease problem,” we created fund decision scenarios by adopting the same paradigm related to monetary incentives as follows (see the full cover story in the Methods section). First, examine both fund-related decisions, and then indicate the following option that you prefer. Gain frame: a sure option (A): a sure gain of ¥600; a gamble option (B): a 30% chance to gain back ¥2,000, and a 70% chance to gain nothing. Loss frame: a sure option (C): a sure loss of ¥1,400; a gamble option (D): a 70% chance to lose ¥2,000, and a 30% chance to lose nothing. In their previous monetary task, 84% of the participants would choose Option A over B in a gain frame, while 87% of the participants would choose Option D over C in a loss frame (Tversky and Kahneman, 1981). Similarly, in monetary gambling tasks, individuals also exhibit more risk-averse behavior when the option is framed as gains rather than framed as losses, independent of the probabilities and reward amounts (De Martino et al., 2006).

Prospect theory proposes that the framing effect is due to reference dependence (Kahneman, 2003). People value certain gains more than probable gains with the equal or higher expected value (i.e., positively framed); the opposite is true for losses in which a risky prospect is preferred to a riskless prospect of equal expected value (i.e., negatively framed). Dual-process theory assumes that decision-making can be described as a function of the intuitive (Type 1) and deliberative (Type 2) processing modes (Reyna and Brainerd, 2011). Deliberative processing modes, such as “think like a scientist,” which can be explained by eye-tracking measures that require more conscious and calculation-based information processing (more fixations, and even a more complete information search; Horstmann et al., 2009), can reduce the framing effect (Thomas and Millar, 2012), and people can consistently exhibit risk aversion or risk seeking regardless of being faced with losses or gains. Additionally, an intuitive processing mode induced by time pressure can increase the framing effect (Guo et al., 2017).

The presence of social cues, such as a pair of eyes, which involves a third party, can serve as a communicative signal and can implicitly influence information processing. However, to date, we are not aware of empirical research that has examined how being watched may influence one’s financial decision-making process. Based on previous research that being observed increases risk seeking and sensitivity to returns (Smith et al., 2014; Silva et al., 2016), we can infer that being watched may influence decision outcomes by influencing information processing. Being watched provides a sense of being monitored (Gervais and Norenzayan, 2012; Pfattheicher and Keller, 2015), which may facilitate rational choices by encouraging deliberative processing or boost risk seeking by increasing reward sensitivity. Our main aim is to examine “whether” and “how” being watched may influence the decision-making processes and outcomes.

### Cues of Being Watched and Decision-Making

For the first aim, we seek to explore whether being watched influences the framing effect and risk-seeking tendency. Cues of being watched, such as the mere presence of an image of “watching eyes,” can be a weighty factor that nudges people’s choices, thus increasing or decreasing their risk tendency. Eye contact constitutes a powerful cue that captures attention (Böckler et al., 2015), and being watched can potentially alter decisions and actions. **In the interpersonal realm,** people behave differently when they know or believe that their actions are being observed (Risko and Kingstone, 2011). Being the object of social attention (the mere presence of images of faces/eyes) automatically and unconsciously affects people’s performance and interpersonal behaviors by increasing arousal and reputational concerns (Northover et al., 2017; Guterstam et al., 2019). Additionally, an image of “watching eyes” can increase compliance because it prevents the reaction that would be expected if a person received explicit advice from others (Bittner and Kulesz, 2015).

**In the intrapersonal realm**, being watched can serve as a signal of social presence and induce the belief that one is noticed by others (Pfattheicher and Keller, 2015). A previous study found that social presence, such as being observed, can boost risk seeking by increasing reward sensitivity, i.e., striatal sensitivity (Smith et al., 2015; Li et al., 2020). This may be a result of the calculations associated with risk predictions and prediction errors, which are associated with information processing (Smith et al., 2015). However, on the contrary, being watched can also lead to self-regulatory success and thus promote rational decision outcomes, such as healthier food choices (Bittner and Kulesz, 2015). Therefore, we infer that when investors decide which fund to purchase or sell, being watched, which is manipulated by presenting an image of an eye, may increase their risk-seeking tendency and promote consistent or rational decision outcomes (i.e., decrease the framing effect). This may be related to real-life scenarios in which investors make fund purchase decisions while being watched by fund managers, family or friends, or even irrelevant people. Ultimately, the described influences of being watched on risk tendencies and framing might result from changes in general information processing strategies (Smith et al., 2015). However, the question of “how” social cues (i.e., being watched) influence the information processing that underlies decision-making in young and older adults remains unclear.

### Cues of Being Watched and Older Adults’ Decision-Making Process

For the second aim, we specifically attempted to understand the impact of being watched on older adults’ decision-making process. For monetary tasks that investigate the framing effect, the weakened cognitive resources of older adults may cause them to rely less on controlled processing in decision-making, which leads to decision biases, such as becoming more susceptible to the framing effect (Pu et al., 2017). However, when the task is personally relevant (receiving money based on their decision outcomes), older adults’ framing effect is less influenced by a frame, while younger adults still show risk seeking in the loss frame (Mikels and Reed, 2009). These findings suggest that whether the frame matters in older adults also relates to decision scenarios and task characteristics. Once the task constitutes high self-relevance or high emotional arousal, the impacts of framing may decrease because stronger motivation is evoked (Hess, 2014). Similarly, older adults may reduce the framing effect more when being watched since they are more motivated when being watched than when not being watched.

With life-span changes in motivation or resources, older adults also show increased concern for the well-being of others (Cho et al., 2020; Mayr and Freund, 2020; Spreng and Turner, 2021). When related to financial decision scenarios, older adults tend to evaluate the credibility of advertising messages according to peripheral cues (Petty and Cacioppo, 1986; Foy et al., 2016). Additionally, older adults are more likely to trust others. They tend to perceive sellers or persuaders as trustworthy and judge a product according to their impression of the seller rather than the product itself (Castle et al., 2012; Ruffman et al., 2012). Correspondingly, we can infer that older adults may be more concerned about social cues and other people’s attention. Older adults may exhibit higher sensitivity to interpersonal cues, particularly eyes that convey robust social and emotional saliency. Compared to younger adults, older adults’ vulnerability to the environment and their sensitivity to socioemotional information (Charles and Carstensen, 2010) may partly explain the different effects of being watched. Consequently, if older adults place greater reliance on contextual information, then their sense of being monitored will increase, and they will be prompted to make more rational choices (i.e., higher decision consistency) when being watched (Lindenberger and Mayr, 2014).

Nevertheless, it is also possible that increased sensitivity to social cues may not be beneficial for older adults. Older adults are less capable of inhibiting the influence of irrelevant information (e.g., social cues) due to cognitive deterioration (Salthouse and Meinz, 1995). Being watched may occupy cognitive resources, thereby increasing risk-seeking behaviors (Conty et al., 2010; Madan et al., 2015). When concerned about decision-making, older adults show diminished sensitivity to social threats or potential losses in social decision-making, such as the Prisoner’s Dilemma Game (PDG; Huang et al., 2020). Being watched may even exert negative influences on older adults’ ability to make rational choices. To date, however, previous findings have produced mixed results. We thus hypothesize that, although they tend to avoid losses, the sociomotivational desire to prioritize social information together with the persuasive power of social attention may prompt older adults to be more susceptible to watching eyes, and they will surrender to social cues more easily than younger adults; thus, their decision outcomes might either be beneficial and promote rational decision outcomes or diminish their sensitivity to losses, which boosts risk seeking.

### Present Research

Accordingly, the empirical findings concerning the mechanism of the watching eyes phenomenon are unclear. Our focus in this study was the impact of being watched, which was presented by an image of eyes, on fund decision-making and risk-seeking behavior in older adults. As few studies have directly empirically tested age differences in this effect, it remains unclear whether the impact of watching eyes, indeed, differs between younger and older ages. Therefore, we also included younger adults as a comparison. We predicted that the presence of an image of “watching eyes” would influence the decision-making process (*via* eye-tracking measures) differently in younger and older adults.

To create a more naturalistic setting, we formulated a fund investment portfolio and described fund fluctuations as losses and gains that are pervasive in fund operation. We examined the framing effect and risk tendency when being watched combined with a gain/loss frame. The participants were considered to exhibit a risk-seeking tendency when they chose to maintain their share in the depressed fund market, which meant either winning or losing the entire initial amount. The participants were considered to exhibit risk avoidance when they withdrew a certain portion (shown as either a gain or a loss) of the funds. To understand the role of information processing when making decisions, eye-tracking techniques were used to reveal the decision makers’ attention allocation to information presented under a gain or loss frame in a natural and relatively non-intrusive way (Orquin and Mueller Loose, 2013; Scholz et al., 2015; Rahal and Fiedler, 2019). Eye-movement measures, such as the number of fixations and total dwell time, were chosen as cognitive effort indices (Horstmann et al., 2009; Kuo et al., 2009). We expected to discover how attention allocation plays a role in the decision-making process.

Based on previous studies, we posit the following hypotheses.

***Hypothesis 1***: Older adults exhibit robust framing effects. Specifically, due to their higher loss-avoidance tendency, they will be more risk seeking when in a loss frame than in a gain frame. For the process measures, more cognitive effort will be invested when in the loss frame for both younger and older adults, as exhibited by more fixations and longer dwell times. However, older adults are, generally, more risk averse than younger adults.

***Hypothesis 2***: The presence of images of “watching eyes” may prompt older adults to keep their money in the depressed fund market even when faced with risks, which causes them to exhibit a risk-seeking tendency. Note that, in the current paradigm, keeping the share in the depressed fund market is considered risk seeking.

***Hypothesis 3***: Frames may interact with the presence of “watching eyes” to influence choices. Since older adults tend to prioritize social information, they are also more susceptible to the frame and may be more susceptible to watching eyes. Thus, compared to younger adults, older adults may reduce the framing effect more when being watched since they are more motivated when being watched than when not being watched.

## Method

### Participants

We determined our sample size through a power analysis performed by using G*power 3.1 (Faul et al., 2007; Best and Charness, 2015; Northover et al., 2017), which indicated that we should include 122 participants for a multivariate analysis of variance (MANOVA) with repeated-measures within-between the interaction for 80% power and an alpha level of 0.05 to detect a medium effect size (*w* = 0.21). We anticipated some missing data due to tracker loss. Thus, to ensure the quality of the eye-tracking data, we oversampled to ensure sufficient minimum sample sizes for each age group. Sixty-seven younger adults were recruited through online posters at Beijing Normal University and nearby universities. Seventy older adults were community residents recruited from communities in the districts of Beijing through online posters and the laboratory’s database. After the experiment, the data from seven older and one younger participants were excluded from the analyses because the participants did not meet the dementia standard on the Clock Drawing Test (CDT; Nolan and Mohs, 1994), failed the eye-movement calibration test, could not follow the instructions or because the data could not be recorded due to experimental errors. Valid data were acquired for the remaining 66 younger adults (40 females; mean age: 23.45 ± 2.65; aged 19 to 30 years) and 63 older adults (33 females; mean age: 65.24 ± 4.02; aged 60 to 81 years), all of whom had fund investment experience (using a one-item question on a six-point scale in which higher scores reflected greater experience; for details, see section “METHOD”). The older adults were randomly assigned to either the eye condition (presented with an image of watching eyes; *n* = 33, 18 females, mean age: 64.85 ± 3.38, aged 60 to 72 years) or the control condition (presented with a neutral image; *n* = 30, 15 females, mean age: 65.67 ± 4.64; aged 60 to 81 years). Likewise, the younger adults were randomly assigned to either the eye condition (*n* = 33, 17 females, mean age: 23.24 ± 2.75; aged 19 to 30 years) or the control condition (*n* = 33, 23 females, mean age: 23.67 ± 2.57; aged 20 to 29 years). The participants had normal or corrected-to-normal vision (Bach, 2006) and completed the study individually. All the participants were compensated with 100 Chinese yuan (approximately $14). The ethics committee of the Faculty of Psychology of Beijing Normal University approved this study.

### Design

This study was a 2 (age group: younger, older) × 2 (condition: eye condition, control condition) × 2 (frame type: gain, loss) mixed design, with the frame type treated as a within-subject variable and age group and eye condition as between-subject variables. The dependent measures were choice data and eye-tracking data. The eye-tracking measures included the fixations per word (including fixations longer than 80 ms and less than 1,200 ms) and dwell time per word [ms; the total time spent within an area of interest (AOI)].

**FIGURE 1:**
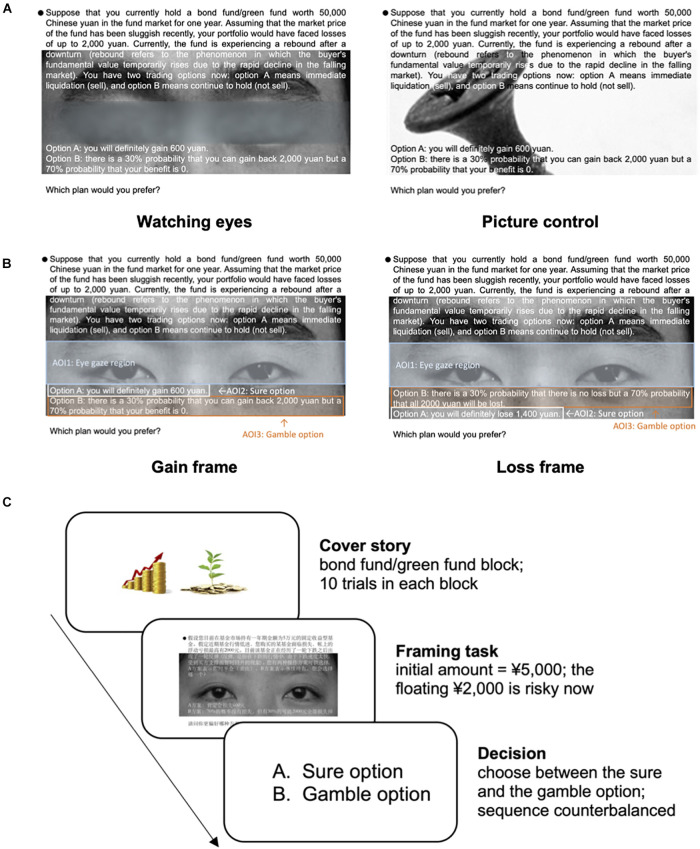
A decision scenario sample, an area of interest (AOI) segment, and a task procedure. **(A)** An example of a decision scenario of two different experimental conditions presented on E-Builder. The background picture changed every trial to capture the participants’ attention. The sequence of the sure and gamble options was counterbalanced. **(B)** AOI segment. The left panel shows the gain frame, and the right panel shows the loss frame. Since the AOI differed between the sure and gamble options, to ensure fairness, we divided the number of fixations and total dwell time by the word count. **(C)** The diagram of the fund decision-making task. The participants were told that they would first receive an initial amount (¥50,000) in the fund market. Assuming that the fund has been sluggish recently, they would not be able to retain a portion of the initial amount (¥2,000) and would have to choose between a sure and a gamble option.

In the experimental condition (eye condition), an image of watching eyes was presented in the background of the decision tasks (see the decision layout in Figure 1A). Similar social presence cues have been used in previous research (e.g., Bateson et al., 2006; Bittner and Kulesz, 2015). However, instead of using a single-eye picture or the eye of Horus, we improved the paradigm by alternating eye pictures in each trial to better capture the participants’ attention (see the AOI segment in Figure 1B and the procedure in Figure 1C). We selected neutral facial expressions from the Chinese Facial Affective Picture System (CFAPS, Gong et al., 2011) to reduce the risk of accidentally priming emotions. Correspondingly, in the control condition, ten non-facial neutral pictures from the International Affective Picture System (IAPS, Bradley and Lang, 2017) were presented, such as households, insects, and cityscapes, which were chosen according to the rating scores from previous research (Xiao et al., 2014; Gong and Wang, 2016) (see Rating Scores in section “Materials”).

### Materials

#### Framing Task

In the modified Asian disease problem (Tversky and Kahneman, 1981), participants receive a virtual financial endowment in a fund market. In the financial-based risky-choice framing paradigm in this study, the participants were presented with a virtual financial endowment at the beginning of each frame type. They were informed that the market price of the fund had been sluggish recently and that the fund that they bought had faced losses. The participants were then asked to choose between taking a certain portion (shown as either a gain or a loss) of the funds or gambling to either win or lose the entire initial amount (see section “Decision Task” in the Supplementary Materials). The floating loss on the account ranged from ¥600 to ¥1,400 in increments of ¥200 to allow for the assessment of framing bias in both small and large amounts (¥1,000 ≈ $156). The participants were then asked to choose between a sure option (guaranteed gain/loss, always Option A) versus a gamble option (risk of gain/loss, always Option B). For the trials framed as a gain, sure options were presented as an option to “gain back” a portion of the initial endowment of money (e.g., “Gain back ¥1,400” of the initial ¥2,000). The loss trials were presented as a sure option to “lose” a portion of the initial endowment of money (e.g., “Lose ¥600” of the initial ¥2,000). The alternative option was to gamble and to risk either gaining or losing the entire initial endowment. Regardless of which option the participants chose, no feedback was supplied.

The participants completed twenty decision tasks (ten gain-framed and ten loss-framed tasks) in which they were asked to choose between sure and gamble options that did or did not have the image of watching eyes as the manipulation. The twenty decision tasks were shown in a completely randomized order. The presentation order of the option (i.e., the gamble option on the top or the bottom of the screen) was changed between trials to avoid the possible influence of the reading order. Interest areas were constructed (as shown in Figure 1B). For clarity, the results were presented as the percentages of risky choices, termed *risk preference*, which can describe one’s tendency to choose a risky or less-risky option. Following this thread of logic, risk seeking means a preference to choose gamble options, while risk averse means a preference to choose sure options.

Since the lengths of the options were different under the gain/loss frames, the areas of the sure option (AOI2: 22,000 mm^2^) and the gamble option (AOI3: 44,500 mm^2^) in the gain frame and the areas of the sure option (AOI2: 19,000 mm^2^) and the gamble option (AOI3: 45,250 mm^2^) in the loss frame were not identical. To control for confounding factors, we divided the eye-tracking measures of each region by the word count (Kuo et al., 2009). Then, the eye-tracking measures to measure information processing included the fixations per word and dwell time per word (ms; the total time spent within an AOI). After the experiment, we constructed these AOI sets for each trial.

#### Eye Pictures

Eye pictures from five males and five females were selected from the neutral facial expressions of the Chinese Affective Picture System (CFAPS; Gong et al., 2011) to reduce the risk of accidentally priming emotions. We cropped the pictures and kept the eye region only. The pixels, brightness, and contrast of the eye pictures were processed uniformly. After unified processing, the pictures were all converted to black and white, 250 × 100 pixels, with horizontal and vertical resolutions of 96 dpi. Before the formal experiment, we had recruited 34 younger and 30 older adults to evaluate the valence and arousal of the pictures by using the 9-point Self-Assessment Manikin Scale (SAM; Bradley and Lang, 1994; Backs et al., 2005; valence: 1 = very negative, 9 = very positive; arousal: 1 = very calm, 9 = very active). Then, we selected eye pictures that were neutral (older: valence: *M* ± *SD* = 4.66 ± 0.36, arousal: *M* ± *SD* = 4.25 ± 0.17; younger: valence: *M* ± *SD* = 4.68 ± 0.41 arousal: *M* ± *SD* = 3.29 ± 0.36).

Correspondingly, in the control condition, we chose ten neutral non-facial pictures from the International Affective Picture System (IAPS; Lang et al., 2008) by using rating scores from previous research (older: valence: *M* ± *SD* = 4.98 ± 0.31, arousal: *M* ± *SD* = 3.92 ± 0.49; younger: valence: *M* ± *SD* = 4.67 ± 0.39, arousal: *M* ± *SD* = 3.84 ± 0.56).

#### Measures

The background measurements comprised the *Positive and Negative Affect Schedule* (PANAS) to measure the participants’ baseline emotional states (Watson et al., 1988); the WAIS-III *vocabulary* subset was administered as a proxy to determine word knowledge and educational achievement, and the WAIS-III *information* subset was also a reflection of acquired knowledge (WAIS-III; Wechsler, 1997). Additional background measurements included *mental arithmetic* and *numeracy* to measure the basic numeric ability needed in fund operation (Weller et al., 2013), *subjective and objective fund experience*, which probed the participants’ experience in or knowledge of fund investment (Li et al., 2015), *negative emotions about financial decisions* (Eberhardt et al., 2019), and *financial risk taking*, which was adopted from the investment subscale in the Domain-Specific Risk-Taking Scale (DOSPERT; Wang et al., 2016) (see Descriptive Statistics in Table 1).

**TABLE 1:** Demographics of the sample.

	Younger (*N* = 66)		Older (*N* = 63)		Age differences	
	Eye	Control	Eye	Control	*t* (127)	*p*
Age (years)	23.24 ± 2.75	23.67 ± 2.57	64.85 ± 3.38	65.67 ± 4.64	69.37	***
Education (years)	16.36 ± 1.67	16.88 ± 2.00	11.3 ± 2.24	11.4 ± 2.51	14.18	***
Monthly household income (thousands)	11.44 ± 6.59	16.93 ± 34.05	9.05 ± 4.78	8.79 ± 4.46	1.70	0.09
Self-rated health	3.94 ± 0.70	3.85 ± 0.62	3.64 ± 0.65	3.9 ± 0.66	1.13	0.26
Visual acuity (decVA)	0.75 ± 0.02	0.75 ± 0.01	0.66 ± 0.10	0.69 ± 0.07	6.52	***
Visual acuity (logMAR)	0.12 ± 0.02	0.12 ± 0.02	0.18 ± 0.07	0.16 ± 0.04	6.07	***
Contrast (logCS)	1.86 ± 0.13	1.89 ± 0.15	1.65 ± 0.21	1.71 ± 0.20	6.32	***
PANAS_Positive	2.88 ± 0.84	2.96 ± 0.66	3.5 ± 0.79	3.56 ± 0.86	4.40	***
PANAS_Negative	1.31 ± 0.32	1.22 ± 0.26	1.44 ± 0.61	1.67 ± 0.66	3.22	**
Vocabulary (WAIS-III)	17.73 ± 1.79	17.18 ± 2.13	14.79 ± 3.26	15.83 ± 2.05	5.13	***
Information (WAIS-III)	22.35 ± 3.31	21.14 ± 4.32	19.89 ± 4.69	20.38 ± 4.76	2.14	*
Mental arithmetic	39.88 ± 9.27	44.97 ± 7.59	23.67 ± 5.84	23.5 ± 7.56	13.68	***
Numeracy	6.06 ± 1.22	6.06 ± 1.48	4.52 ± 1.09	4.9 ± 1.00	6.37	***
Subjective fund experience	4.18 ± 0.95	3.91 ± 0.95	4.64 ± 0.70	4.73 ± 0.58	4.47	***
Objective fund experience	2.85 ± 0.76	2.67 ± 0.54	2.55 ± 0.67	2.47 ± 0.63	2.18	*
Negative emotions about financial decisions	1.72 ± 0.59	1.88 ± 0.74	1.8 ± 0.65	1.86 ± 0.65	0.28	0.78
Financial risk-taking	4.26 ± 1.10	4.36 ± 1.13	4.06 ± 1.07	4.42 ± 0.65	0.45	0.65

Monthly household income is expressed in units of 1,000 yuan (RMB). Self-rated health was assessed using a 5-point scale; higher scores indicated better health status. PANAS represents the Positive and Negative Affect Schedule used to measure the participants’ baseline emotional experiences. WAIS-III represents the Wechsler Adult Intelligence Scale, 3rd edition. The asterisks indicate significant differences between older and younger adults (independent sample t-test). *p < 0.05, **p < 0.01, and ***p < 0.001.

#### Apparatus

Stimuli were presented by using the SR Research Experiment Builder. An Eyelink 1000 plus eye tracker (SR Research, Mississauga, ON, Canada) was used to record the eye-movement data during the decision-making process. We tracked the participants’ eye movements with the combined pupil and corneal reflection setting at a sampling rate of 1,000 Hz (right eye only). Head movements were tracked, although we used a chin rest to minimize head movement. The participants indicated all their responses by pressing a button on a standard QWERTY keyboard. The screen resolution was 1,680 × 1,050, and the viewing angle of the stimulus material was 28.7 degrees horizontally and 22.9 degrees vertically (a 19-inch monitor; a screen ratio, 5:4; eyes, 70 cm from the screen). AOIs were defined by using Data Viewer.

#### Manipulation Check

To check whether the participants paid attention to the image of watching eyes while making decisions, we computed the fixations on the eye gaze region (AOI1). We also asked them to rate the pictures’ valence and arousal after the task using the SAM. The results of the indicators mentioned are shown and explained below. We also checked the fixations on the eye gaze region (AOI1) to examine whether the participants were aware of the eyes (see Results).

### Procedure

The study involved a single, 2-h-long experimental session. Each participant was asked to complete two tasks. The first task was completed by using a computer and an Eyelink 1000 system, while the second task asked the participants to complete a paper-and-pencil questionnaire. After completing the written consent form and the demographic questionnaire (including age, years of education, monthly household income, and self-rated health status), the participants completed the CDT (the younger adults skipped this step) to screen for possible symptoms of dementia. Before the eye-tracking experiment, we had administered the PANAS for the baseline emotional states, and then the experimenter explained the principle and the process of the eye-movement investigation to relax the participants. We used FrACT to measure the participants’ vision to ensure that their eyesight was normal or corrected to normal and that they had no eye diseases, such as glaucoma and cataracts. A chinrest was adjusted accordingly, and the distance between the participants’ eyes and the screen was 70 cm.

The participants were first presented with two decision trials to familiarize them with the procedure; then, the experiment began. This design was used to ensure that the frame, the specified amount, or the probability of gain experienced in the practice trial would not influence the participants. Then, the participants completed twenty decision tasks with a 10-min break. We changed the eye pictures after each trial to better capture the participants’ attention. The order of the decision scenarios was counterbalanced. The results of their decisions were recorded, but no feedback was given. After the participants completed all decision tasks, background measurements were conducted. The data were analyzed with SPSS 25.0 and R 4.0.3 (R Core Team, 2020).

## Results

We first checked whether the participants noticed the watching eyes *via* the eye-tracking measures. Then, we implemented a series of analyses to examine whether the image of watching eyes affected people’s framing effect and risk preference. Afterward, we explored the underlying mechanism that links being watched to decision outcomes by analyzing the eye-tracking measures. Finally, considering the nested data structure, we executed multilevel modeling (MLM) using the eye-tracking measures to answer how being watched may influence the decision-making process.

### Demographics and Covariables Information

Table 1 presents the demographic information of the sample. No significant differences were found between the two age groups or experimental groups when using a 2 (age group: younger, older)- × –2 (condition: eye condition, control) ANOVA in terms of the *monthly household income*, *self-rated health*, *negative emotions about financial decisions* or *financial risk taking*. However, the younger adults had a significantly higher *educational level*, *F*(1,125) = 199.10, *p* < 0.001, *Mean Difference* (*MD*) = 5.27, *vocabulary*, *F*(1,125) = 26.09, *p* < 0.001, *MD* = 15.31 and *information* score, *F*(1,125) = 4.49, *p* < 0.05, *MD* = 1.60, a richer *objective fund experience*, *F*(1,125) = 4.78, *p* < 0.05, *MD* = 0.25, better *mental arithmetic* ability, *F*(1,125) = 194.70, *p* < 0.001, *MD* = 18.84 and higher *numeracy* scores than the older adults, *F*(1,125) = 39.89, *p* < 0.001, *MD* = 1.35. The older adults reported more intense *positive*, *F*(1,125) = 19.18, *p* < 0.001, *MD* = 0.61, and *negative emotions at the baseline* than the younger adults, *F*(1,125) = 11.31, *p* < 0.01, *MD* = 0.29. Additionally, the older adults were more experienced in fund investment (*subjective fund experience*), *F*(1,125) = 19.79, *p* < 0.001, *MD* = 0.64.

### Manipulation Check

To check whether the participants noticed the pictures presented during the task, we computed the fixations on the eye gaze region (AOI1) *via* eye-tracking measures (older: gain frame: *M* ± *SD* = 1.88 ± 2.61, loss frame: *M* ± *SD* = 1.91 ± 2.44; younger: gain frame: *M* ± *SD* = 1.84 ± 2.38, loss frame: *M* ± *SD* = 2.41 ± 3.76). The results of the *t*-tests showed that the participants were attentive to the eye pictures (all *p*s < 0.001) compared to no fixations.

We also carried out the 2 (age: younger, older)- × –2 (condition: eye condition, control) ANOVAs on ratings of valence and arousal of eye gaze and neutral pictures. Results found no age group main effect, condition main effect, nor age × condition interactions. As for valence, results found no significant age group main effect, condition main effect, nor age × condition interactions (*p*s > 0.05; eye gaze pictures: older: *M* ± *SD* = 4.72 ± 0.72, younger: *M* ± *SD* = 4.38 ± 0.82; neutral pictures: older: *M* ± *SD* = 4.79 ± 1.10; younger: *M* ± *SD* = 4.44 ± 0.99). As for arousal, results also found no significant age group main effect, condition main effect, nor age × condition interactions (*p*s > 0.05; eye gaze pictures: older: *M* ± *SD* = 4.08 ± 0.55, younger: *M* ± *SD* = 4.14 ± 0.52; neutral pictures: older: *M* ± *SD* = 4.37 ± 1.22; younger: *M* ± *SD* = 4.20 ± 0.78).

### Risk Preference Under the Gain/Loss Frame in Decision-Making

**FIGURE 2:**
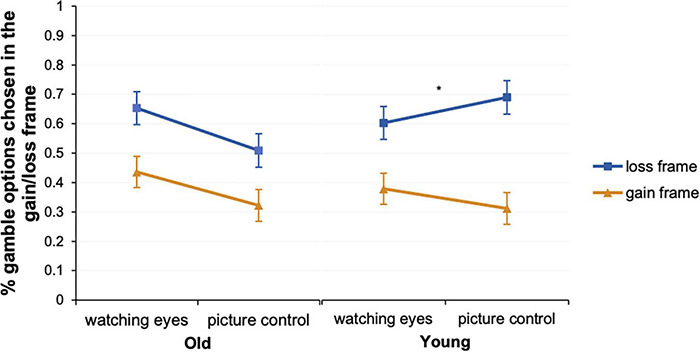
Percentages of the gamble choices in younger and older adults under the gain/loss frame in the eye and control conditions. The error bars indicate the standard error of the mean. The asterisk indicates a significant difference in the frame by watching eye interactions (repeated-measures ANOVA). *p < 0.5.

To determine whether the framing effect exists (***Hypothesis 1***) and whether being watched prompts risk seeking (***Hypothesis 2)***, we used a 2 (age group: younger, older) × 2 (condition: eye condition, control) × 2 (frame: gain, loss) ANOVA (see Table 2 and Figure 2). Consistent with ***Hypothesis 1***, the **main effect of a frame** revealed that the participants were susceptible to the frame, *F*(1,125) = 79.99, *p* < 0.001, η^2^ = 0.39, in that they exhibited riskier behavior in the loss frame (*M* = 0.63 ± 0.02) than in the gain frame (*M* = 0.61 ± 0.04). The main effects of the **age group** and **eye gaze** were not significant (*p*s > 0.05).

**TABLE 2:** Descriptive statistics for the decision outcomes and eye-tracking measures.

			Younger (*N* = 66)		Older (*N* = 63)	
			*M*	*SD*	*M*	*SD*
**Choice percentage (%)**						
Gain frame	watching eyes	sure option	0.62	0.25	0.56	0.31
		gamble option	0.38	0.25	0.44	0.31
	control	sure option	0.69	0.24	0.68	0.28
		gamble option	0.31	0.24	0.32	0.28
Loss frame	watching eyes	sure option	0.40	0.29	0.35	0.28
		gamble option	0.60	0.29	0.65	0.28
	control	sure option	0.31	0.22	0.49	0.31
		gamble option	0.69	0.22	0.51	0.31
**Fixations**						
Gain frame	watching eyes	sure option	0.60	0.18	1.03	0.32
		gamble option	0.48	0.16	0.83	0.33
	control	sure option	0.67	0.27	1.22	0.49
		gamble option	0.53	0.24	0.98	0.51
Loss frame	watching eyes	sure option	0.81	0.32	1.23	0.56
		gamble option	0.56	0.28	0.91	0.42
	control	sure option	0.80	0.32	1.48	0.67
		gamble option	0.60	0.23	1.06	0.51
**Dwell time**						
Gain frame	watching eyes	sure option	128.10	44.03	241.57	68.37
		gamble option	100.34	41.82	193.86	73.99
	control	sure option	141.75	58.55	296.79	112.31
		gamble option	107.54	51.16	239.65	129.41
Loss frame	watching eyes	sure option	171.93	68.07	292.84	120.58
		gamble option	116.70	60.58	212.93	95.13
	control	sure option	141.75	58.55	343.81	152.65
		gamble option	107.54	51.16	266.00	138.85

Two marginally significant results were found, and a simple-effect analysis was conducted for exploratory reasons. **Age group × eye interactions** were marginally significant, *F*(1,125) = 3.20, *p* = 0.076, η^2^ = 0.03. This is consistent with ***Hypothesis 2***, which indicates that older adults are more prone to seek risks when gazed at by a pair of eyes, *F*(1,125) = 5.38, *p* < 0.05, η^2^ = 0.04; *MD* = 0.13, but younger adults are not affected (*p* = 0.85). Additionally, marginally significant **age group × frame interactions** were found, *F*(1,125) = 3.09, *p* = 0.08, η^2^ = 0.02, which demonstrates that the influence of frame on risk preference is more robust in the younger adults than in the older adults [for the older adults, *F*(1,256) = 25.20, *p* < 0.001, η^2^ = 0.17, *MD* = 0.20, and for the younger adults, *F*(1,256) = 58.79, *p* < 0.001, η^2^ = 0.32; *MD* = 0.30]. In the gain frame, there were no significant differences found between the older and younger adults, *F*(1,125) = 0.49, *p* = 0.48, η^2^ = 0.004; *MD* = 0.03, while the younger adults exhibited higher risk preference in the loss frame, *F*(1,125) = 1.83, *p* = 0.18, η^2^ = 0.01; *MD* = 0.07. ***Hypothesis 3*** was not supported since we did not find age group × frame × eye interactions. No other significant main or interactive effects were found.

The results showed no age group differences in the risk percentage and no significant interactions between the age group and eye condition or framing (only marginal). However, as we were mainly interested in how cues of being watched influence the decision outcomes of both age groups, we then **explored** the effects of framing and the eye gaze condition for the two age groups separately. Given that there were no significant interactions in the main analyses, the results concerning the age group differences cannot be generalized to the general population and require further replication.

To detect the effect of an image of watching eyes on risk preference while allowing for an effect of the comparison within different age groups, we further conducted two two-way ANOVAs for each age group. When we compared the effect of being watched separately, an interesting pattern emerged. For the older adults, in addition to the **main effect of a frame**, *F*(1,61) = 19.81, *p* < 0.001, η^2^ = 0.25, a significant **main effect of eye** revealed that they were more likely to engage in risky behavior when gazed at by a pair of eyes, *F*(1,61) = 4.82, *p* < 0.05, η^2^ = 0.07, *MD* = 0.13. This finding corroborates ***Hypothesis 2*** that older adults are more prone to seek risks when gazed at by a pair of eyes. They made more risky choices in the eye condition (*M* = 0.55 ± 0.04) than in the control condition (*M* = 0.42 ± 0.04). We additionally controlled for education, which was the only demographic variable that correlated with risk preference (*r* = 0.30, *p* < 0.05). The **eye** effect remained significant, *F*(1,60) = 5.33, *p* < 0.05, η^2^ = 0.08, and even increased in strength, which suggests that the observed findings cannot be simply explained by individual differences in education. However, the main effect of frame disappeared (*p* = 0.79), which is consistent with previous findings that decision rationality is positively related to the education level (Fan, 2017).

For the younger adults, in addition to the **main effect of a frame**, *F*(1,64) = 79.26, *p* < 0.001, η^2^ = 0.55, we observed significant **frame × eye interactions,** *F*(1,64) = 5.16, *p* < 0.05, η^2^ = 0.08, which indicates that they are less susceptible to the frame when gazed at by a pair of eyes (eye: *MD* = 0.22, *p* < 0.001, η^2^ = 0.49; control: *MD* = 0.38, *p* < 0.001, η^2^ = 0.26), as indicated by the smaller effect size. Cues of being watched decreased younger adults’ decision bias by weakening the role of the frame but had no influence on risk seeking. Accordingly, ***Hypothesis 3*** was not supported among the older adults. However, aligned with the analysis above, it seems that the younger adults’ framing effect was reduced when they were gazed at by eyes.

### Eye-Tracking Analysis in Decision-Making

**FIGURE 3:**
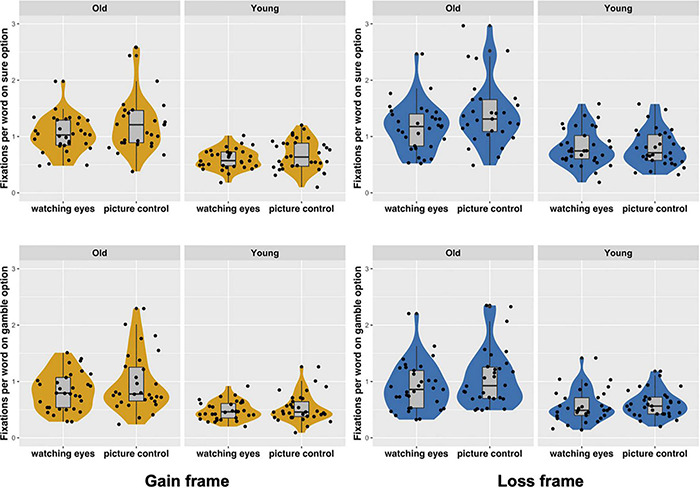
Violin plots of the eye-tracking measures. Each black dot indicates the fixations per Hanzi of one individual. The shaded yellow area depicts the eye-movement distribution within the gain frame, whereas the blue area depicts the eye-movement distribution within the loss frame. The panel represents the number of fixations per word on the sure or gamble option under the gain/loss frame. The upper two plots show fixations on the sure option, whereas the lower two plots show fixations on the gamble option.

The eye-movement measures (the number of fixations and dwell time per word) based on the AOI were analyzed as indices of the cognitive effort and attention allocated toward the sure and gamble options when making decisions (see Table 2 and Figure 3). Since the AOIs for the sure and gamble options differed, to ensure fairness, we divided the number of fixations and total dwell time by the Hanzi word count (Kuo et al., 2009). To ensure the quality of the eye-tracking data, we excluded trials in which tracker loss occurred and any single fixation duration that was less than 80 ms or greater than 1,200 ms by using Data Viewer (Rayner, 2009).

We first conducted a 2 (age group: younger, older) × 2 (condition: eye condition, control) × 2 (frame: gain, loss) × 2 (option: sure, gamble) ANOVA. The results found that the main effect of **age group** revealed that there were more fixations among older adults, *F*(1,124) = 57.71, *p* < 0.001, η^2^ = 0.32; *MD* = 0.44, the main effect of **frame** indicated more fixations under the loss frame, *F*(1,124) = 32.72, *p* < 0.001, η^2^ = 0.21; *MD* = 0.14, and the main effect of **option** showed more fixations on sure options *F*(1,124) = 261.76, *p* < 0.001, η^2^ = 0.68; *MD* = 0.24. Interactions were found to be significant on **age group × option**, *F*(1,124) = 19.71, *p* < 0.001, η^2^ = 0.14, which indicates that both the older and younger adults’ tendency to attend to the sure option was stronger than that of the gamble option (old: *MD* = 0.31, *p* < 0.001, η^2^ = 0.62; young: *MD* = 0.18, *p* < 0.001, η^2^ = 0.37). Additionally, the interactions on **frame × option** were found to be significant, *F*(1,124) = 16.71, *p* < 0.001, η^2^ = 0.12, which suggests that there were more fixations on the sure option under the loss rather than the gain frame, particularly on the sure-loss option (sure: *MD* = 0.20, *p* < 0.001, η^2^ = 0.23; gamble: *MD* = 0.10, *p* < 0.001, η^2^ = 0.10).

Following the logic of the risk preference data analysis, we then conducted an exploratory analysis on the effects of framing and the eye gaze condition on the fixation patterns for the two age groups separately. Two 2 (condition: eye condition, control) × 2 (frame: gain, loss) × 2 (option: sure, gamble) ANOVAs were performed separately for the two age groups to examine the effect of eye gaze, a frame, and an option on the fixations per word. For the older adults, the main effects of **frame**, *F*(1,60) = 11.91, *p* < 0.01, η^2^ = 0.17; *MD* = 0.15, **option**, *F*(1,60) = 143.60, *p* < 0.001, η^2^ = 0.71; *MD* = 0.31, and **frame × option interactions**, *F*(1,60) = 7.54, *p* < 0.05, η^2^ = 0.11 were found, which suggests that there were more fixations under the loss frame on the sure option, particularly on the sure-loss option. Marginally significant **option × eye interactions** were also found, *F*(1,60) = 3.80, *p* = 0.056, η^2^ = 0.06, which show that the older adults were less attentive to both options in the eye condition (eye: *MD* = 0.26, *p* < 0.001, η^2^ = 0.47; control: *MD* = 0.36, *p* < 0.001, η^2^ = 0.60). The decrease was greater for the sure option, as indicated by the greater mean difference (sure: *MD* = 0.20, *p* < 0.10, η^2^ = 0.05; gamble: *MD* = 0.10, *p* > 0.05, η^2^ = 0.02). Eye presence may influence the processing of the sure option. However, the attention allocated to the gamble option was maintained.

Generally, the image of watching eyes did not significantly impact the eye movement of younger adults. However, similar to the older adults, we also found **frame,** *F*(1,64) = 30.66, *p* < 0.001, η^2^ = 0.32; *MD* = 0.12, and **option,** *F*(1,64) = 119.89, *p* < 0.001, η^2^ = 0.65; *MD* = 0.18, main effects and **frame × option interactions**, *F*(1,64) = 11.24, *p* < 0.01, η^2^ = 0.15, which indicate that the younger adults’ tendency to attend to the sure-loss option was also stronger (see Figure 3).

An analysis of the differences in the dwell times per word between the sure and gamble options revealed a similar tendency, although we did not find an interaction between the option and eye conditions among the older adults as the result of fixations per word.

### The Underlying Mechanism of Watching Eyes on Decision Outcomes

To explore the possible relationship among the eye gaze, risk preference, and eye-tracking measures, while taking into consideration individual-level differences and the nesting data attributes, we performed MLM with the *lme4* and *lmerTest* packages for R (Bates et al., 2015) and organized the results with *bruceR* (Bao, 2021). As part of the first step in the model building process, we computed the intraclass correlation [ICC = τ00/(τ00 + σ2)], which quantifies the proportion of the total variation in trials (i.e., manipulations) accounted for by individual differences. The results showed that differences across the participants accounted for approximately 38.89% of the variance in the first level (the null model). This variance pattern necessitated the use of MLM to control for these differences (Hox et al., 2017).

To estimate how watching eyes influenced the decision outcomes, we nested the risk preference under different frame types (Level 1) within the participants (Level 2). We computed three models: a baseline model that included an intercept and the dummy indicator of the frame type and the option, both random at Level 2 (Model 1); a constrained model with the addition of the fixed effects of the proposed predictors (age group, eye, and fixations) that predicted Level 2 variation in the intercept (Model 2), which means that the predictors were free to vary independently; and a model that contained an interaction between the eye and the option found in the previous ANOVA results (the Interaction Model).

The results showed a highly significant reduction in deviance compared with the null model. Model 2 revealed an increased significant influence of frame [β = –0.252, *SE* = 0.017, *t*(406) = –14.67, *p* < 0.001, 95% CI = (−0.29, −0.22)] on risk preference (see Table 3). The models suggested the unique contributions of the loss frame to increased risk preference. In contrast, Akaike’s information criterion (AIC) showed that the contribution of the interaction between the image of watching eyes and the option did not significantly improve the model fit, and the interaction was non-significant. Additionally, the eye-tracking measures (fixations) were unrelated to risk preference (see Table 3).

**TABLE 3:** A summary of multilevel regression models predicting the risk preference of process variables (Level 1: *n* = 516) nested within participants (Level 2: *n* = 129) with separate random intercepts.

	Null model	Model 1	Model 2	Interaction Model
Predictors	*B* (β)	*B* (β)	*B* (β)	*B* (β)
(Intercept)	0.490*** (0.020)	0.616*** (0.023)	0.591*** (0.042)	0.591*** (0.043)
frame		−0.253*** (0.016)	−0.252*** (0.017)	−0.252*** (0.017)
option		0.000 (0.016)	−0.001 (0.019)	−0.001 (0.025)
fixations			0.004 (0.036)	0.004 (0.036)
condition			0.058 (0.040)	0.058 (0.043)
age group			−0.016 (0.043)	−0.016 (0.043)
condition × option				−0.000 (0.033)
Marginal R^2^	0.000	0.174	0.182	0.181
Conditional R^2^	0.389	0.620	0.623	0.622
AIC	152.222	−16.344	2.684	9.665
BIC	164.960	4.886	36.638	47.862
Num.obs.	516	516	515	515
Num.groups: Subject	129	129	129	129
Var:code(Intercept)	0.036	0.041	0.041	0.041
Var:residual	0.056	0.035	0.035	0.035

The table presents estimates from a multilevel model analysis with decision outcome data nested within participants. Unstandardized regression coefficients are displayed, with standard errors in parentheses. Deviance is calculated as −2 × log-likelihood. The values of β for each parameter are derived from B weights by multiplying the standard deviation of the predictor and dividing the standard deviation of the outcome (Hox et al., 2017). Because between-participant variance was excluded from these analyses by within-participant centering, we used the within-participant standard deviations. ***p < 0.001.

## Discussion

In the current study, the framing effect was found to be robust in both the younger and older adults. We did not find direct evidence that the older and younger adults behaved differently when making fund decisions, and they were not more risk seeking when gazed at by eyes. For the process measures, the eye-tracking results (more fixations and a longer dwell time) revealed that the older adults were more conservative in their processing speed, either to avoid errors, or because the quality of their processing speed was worse, or both.

However, since we were specifically interested in how cues of being watched influence the decision outcomes of both age groups, the exploratory findings suggest that an image of watching eyes primarily increased risk-seeking behavior in the older adults but decreased the decision bias caused by the frame in the younger adults. Although both the younger and older adults exhibited susceptibility to the frame, the younger adults’ risk-seeking tendency was stronger, more so in the loss frame than in the gain frame. The eye-tracking measures indicated that watching eyes resulted in the older adults giving reduced attention to the sure option, thereby escalating their risk seeking. However, for the younger adults, although watching eyes decreased their susceptibility to the frame and facilitated consistency in decision-making, an image of watching eyes seemed to have little influence on their information processing.

### Older and Younger Adults Were Equally Susceptible to the Frame

Both the older and younger adults exhibited a tendency to take risks under the loss frame. However, the older adults were not more risk averse, as predicted by the age differences (***Hypothesis 1*** was partly validated). The life-span perspective holds that older adults are generally oriented toward maintenance and loss avoidance, while younger people strive for gains in the perceived accumulation of resources/assets (Gong and Freund, 2020). Our findings also support the expectations of theories of goal orientation, which suggest a shift away from securing gains in younger adulthood toward maintenance and loss avoidance in older adulthood. From this perspective, the exploratory eye-movement measures also demonstrated that a sure loss captured the most attention in older adults. The eye-tracking findings were consistent with the expectations of prospect theory, which proposes that people are, generally, more sensitive to losses (Pachur et al., 2018; Zeng et al., 2019). Older adults showed increased loss-shift behaviors, which suggests that losses have a more considerable impact than gains on the decision-making behaviors of older adults (Cooper et al., 2017).

### Cues of Being Watched Increased Risk-Seeking Behavior in Older Adults

The behavioral results did not provide direct evidence that eye gaze boosted risk seeking in older age. However, the exploratory results suggested a trend that an image of watching eyes increased risk-seeking behavior in older adults (***Hypothesis 2*** was validated), which can be partly explained by the eye-movement measures. Age-related changes in decision-making have been attributed to the deterioration of cognitive skills. Research has shown age-related decreases in the ability to inhibit goal-irrelevant information in the background (Hadar et al., 2021). Our results also showed that cues of being watched, which are irrelevant to the present task, decreased the processing of the sure option, but the attention given to the gamble option was maintained. We postulated that an image of watching eyes would trigger reward sensitivity (Smith et al., 2015). The older adults maintained their cognitive effort in evaluating the gamble option and calculated the possible consequences if they chose to gamble, which resulted in a greater preference for risk. The decision-making data supported our presumption that so-called risk seeking would result in older adults keeping their share in the fund market even when faced with the risk of loss.

The eye-tracking measures confirmed that maintained attention to the gamble option matters. Additionally, dual processing predicts that individuals who use fast and intuitive processing are likely to show greater framing effects than individuals who use slow and deliberative processing to approach decisions (Perez et al., 2018). The presence of watching eyes seems to increase the deliberative processing on gamble options, which accentuates cognitive effort asymmetry in processing different options and eventually influences the decision outcomes.

Accordingly, it seems that the eye cues subtly affected investment choices by signaling that the decision makers were being watched. However, we did not find an interaction among age, a frame, and the presence of watching eyes (***Hypothesis 3*** was not validated) to influence their choices. The cues of watching eyes can alter one’s attention allocation to potential choices; however, the descriptive frame itself is the most robust, and watching eyes may exert little influence. One possible reason might be that we did not explicitly ask the participants to give attention to the eye gaze background. The eye background can exert only an implicit influence, which is not comparable to a prevalidated robust framing effect.

### Cues of Being Watched Prompted Younger Adults to Act More Rationally

The exploratory findings also suggested a statistical trend that the cues of being watched decreased the younger adults’ decision bias caused by the frame, which increased economic rationality (less influenced by the frame). This finding is consistent with those of previous food decision studies in which eye watching activated self-regulatory success and thus promoted rational decision outcomes (Bittner and Kulesz, 2015).

However, in contrast to previous research that has stated that social presence encourages younger adults to take more risks and prefer immediate rewards and results in heightened activation of the striatum (Smith et al., 2014, 2015; Weigard et al., 2014), we did not find a heightened risk-seeking tendency among the younger adults. Different from previous research that shows that reward sensitivity might be triggered by the presence of a young adult’s friend, we used only anonymous eye pictures. This could be the reason that young adults would not perceive the eye gaze as a cue to increase their perceived value of the risky decision. Peer influence on reward sensitivity among younger adults might depend on their familiarity with the observer. Additional research is needed to demonstrate whether this is a robust result and whether our explanation is viable.

### Limitations, Implications, and Future Directions

First, the current study could not determine the mental process by which the presence of watching eyes influenced fund decision outcomes. Moreover, we did not find a mediation effect of the process measures, i.e., the eye-movement measures, on the relation between the presence of watching eyes and the decision outcomes or cross-level interaction effects by using MLM (Aguinis et al., 2013). Possible reasons could be that the watching eyes phenomenon is too weak to be detected, as found in a previous meta-analysis (Northover et al., 2017) and in our marginally significant interactions. Alternatively, the task was highly personally relevant, which left little space for the participants’ motivation to further improve when they were watched. Although the human perception system is sensitive to social information, repetitive and prolonged exposure can inhibit the effect of watching eyes. People can be habitualized, thereby reducing the sense of being watched (McSweeney and Murphy, 2000; Haley and Fessler, 2005).

Second, the MLM results did not necessarily show contributions of eye-tracking measures to decision outcomes. Consistent with previous research, the eye-movement measures mostly served as indicators of information processing, such as search strategies, cognitive efforts, or arousal (Glöckner and Herbold, 2011; Fiedler and Glöckner, 2012; Rubaltelli et al., 2016; Stewart et al., 2016). One possible reason is that the eye-tracking indices that we used are constrained to cognitive effort measurements.

Third, reading skills, such as reading fluency and reasoning ability, are associated with various eye movement parameters (Benfatto et al., 2016; Thibaut and French, 2016), which might influence eye-fixation patterns during reading. To yield a more robust result, this would be an interesting covariate to control. We note these three points as limitations of the current work and interesting lines for future research.

The findings obtained here are informative in two key aspects. At the theoretical level, we used embedded framed decision tasks in a naturalistic fund investment scheme while allowing decision makers to choose with an image of watching eyes. Compared to canonical research, the investigation of this daily social interaction mechanism is relatively new. At the applied level, understanding this contextual sensitivity is meaningful for developing decision aids to promote rational financial decision making, such as human-robot interactions. Future research should focus on how other social signals, such as interactive gestures or biological motions, play a role in altering personal decisions. Another interesting future research direction would be to examine whether the watching eyes phenomenon can be generalized to real-life decision-making scenarios with interactive eye contact rather than eye pictures. For example, live streamers persuade their customers to buy more through eye contact in online live streaming marketing.

## Conclusion

The current study assessed whether the cues of being watched affect the financial risk preferences of older and younger adults. Framing effects were found in both older and younger adults. We found an inconclusive conclusion that watching eyes increased the reckless gambling behaviors of older adults, which led them to maintain their share in the depressed fund market. Eye-tracking indicators, such as the number of fixations and dwell time, showed that eye presence might influence the processing of the sure option. However, the attention allocated to the gamble option was maintained. The exploratory findings suggested a statistical trend that the cues of being watched decreased the decision bias caused by the verbal framing of younger adults. These findings provide preliminary evidence on how minimal social cues, even the presence of watching eyes, influence risk preferences in fund operation processes for older and younger adults. The results concerning age differences cannot be generalized to the general population and still require further replication.

## Data Availability Statement

The raw data supporting the conclusions of this article will be made available by the authors, without undue reservation.

## Ethics Statement

The studies involving human participants were reviewed and approved by the ethics committee of the Faculty of Psychology from Beijing Normal University. The patients/participants provided their written informed consent to participate in this study.

## Author Contributions

ML: conceptualization, methodology, software, formal analysis, investigation, data curation, writing – original draft, writing – review and editing, and visualization HP: writing – review and editing, supervision, project administration, and funding acquisition. Both authors contributed to the article and approved the submitted version.

## Conflict of Interest

The authors declare that the research was conducted in the absence of any commercial or financial relationships that could be construed as a potential conflict of interest.

## Publisher’s Note

All claims expressed in this article are solely those of the authors and do not necessarily represent those of their affiliated organizations, or those of the publisher, the editors and the reviewers. Any product that may be evaluated in this article, or claim that may be made by its manufacturer, is not guaranteed or endorsed by the publisher.
