# Supplementary material for: How Cues of Being Watched Promote Risk Seeking in Fund Investment in Older Adults
Source: Front Psychol. 2022 Jan 12;12:765632. doi: 10.3389/fpsyg.2021.765632 (PMC8790478; doi:10.3389/fpsyg.2021.765632)
Supplement: Supplementary file 1 [file Data_Sheet_1.pdf]

How Cues of Being Watched Promote Risk Seeking in Fund Investment in Older Adults

Supplementary Materials

Decision task

Cover Story

Green Fund

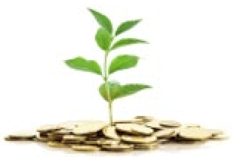

Environmental protection enterprise funds mean that assets are mainly invested in the environmental protection industry. If investors purchase funds, their money will be handled equivalently to investing funds in companies engaged in environmental protection, which will yield stable returns with low risk. The philosophy of the green fund is characterized by energy savings and environmental protection, and this mutual fund is a socially responsible investment that emphasizes the ecological responsibility of the invested company. Investing in such a fund is conducive to environmental protection.

Bond Fund

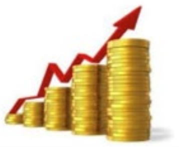

Fixed income funds mean that assets are mainly invested in bonds. If investors purchase funds, their money will be handled equivalently to investing funds in the government, which will yield stable returns with low risk. The philosophy of the bond fund is characterized by increasing stability of income through bond portfolio combinations. This mutual fund can achieve higher yields than depositing existing assets in banks. Investing in such a fund is conducive to gaining asset appreciation.

Framing tasks

Suppose that you currently hold a bond fund/green fund worth 50,000 Chinese yuan in the fund market for one year. Assuming that the market price of the fund has been sluggish recently, your portfolio would have faced losses of up to 2,000 yuan. Currently, the fund is experiencing a rebound after a downturn (rebound refers to the phenomenon in which the buyer's fundamental value temporarily rises due to the rapid decline in the falling market). You have two trading options now: option A means immediate liquidation (sell), and option B means continue to hold (not sell). Which plan would you prefer?

If option A is adopted, you will definitely gain 600 yuan.

If option B is adopted, there is a 30% probability that you can gain back 2,000 yuan but a 70% probability that your benefit is 0.

If option A is adopted, you will definitely lose 1,400 yuan.

If option B is adopted, there is a 30% probability that there is no loss but a 70% probability that all 2000 yuan will be lost.

If option A is adopted, you will definitely gain 800 yuan.

If option B is adopted, there is a 40% probability that you can gain back 2,000 yuan but a 60% probability that your benefit is 0.

If option A is adopted, you will definitely lose 1,200 yuan.

If option B is adopted, there is a 40% probability that there is no loss but a 60% probability that all 2000 yuan will be lost.

If option A is adopted, you will definitely gain 1,000 yuan.

If option B is adopted, there is a 50% probability that you can gain back 2,000

yuan but a 50% probability that your benefit is 0.

If option A is adopted, you will definitely lose 1,000 yuan.

If option B is adopted, there is a 50% probability that there is no loss but a 50% probability that all 2000 yuan will be lost.

If option A is adopted, you will definitely gain 1,200 yuan.

If option B is adopted, there is a 60% probability that you can gain back 2,000 yuan but a 40% probability that your benefit is 0.

If option A is adopted, you will definitely lose 800 yuan.

If option B is adopted, there is a 60% probability that there is no loss but a 40% probability that all 2000 yuan will be lost.

If option A is adopted, you will definitely gain 1,400 yuan.

If option B is adopted, there is a 70% probability that you can gain back 2,000 yuan but a 30% probability that your benefit is 0.

If option A is adopted, you will definitely lose 600 yuan.

If option B is adopted, there is a 70% probability that there is no loss but a 30% probability that all 2000 yuan will be lost.
